# Supplementary figures and images for: CREBBP/EP300 acetyltransferase inhibition disrupts FOXA1-bound enhancers to inhibit the proliferation of ER+ breast cancer cells
Source: PLoS One. 2022 Mar 30;17(3):e0262378. doi: 10.1371/journal.pone.0262378 (PMC8967035; doi:10.1371/journal.pone.0262378)

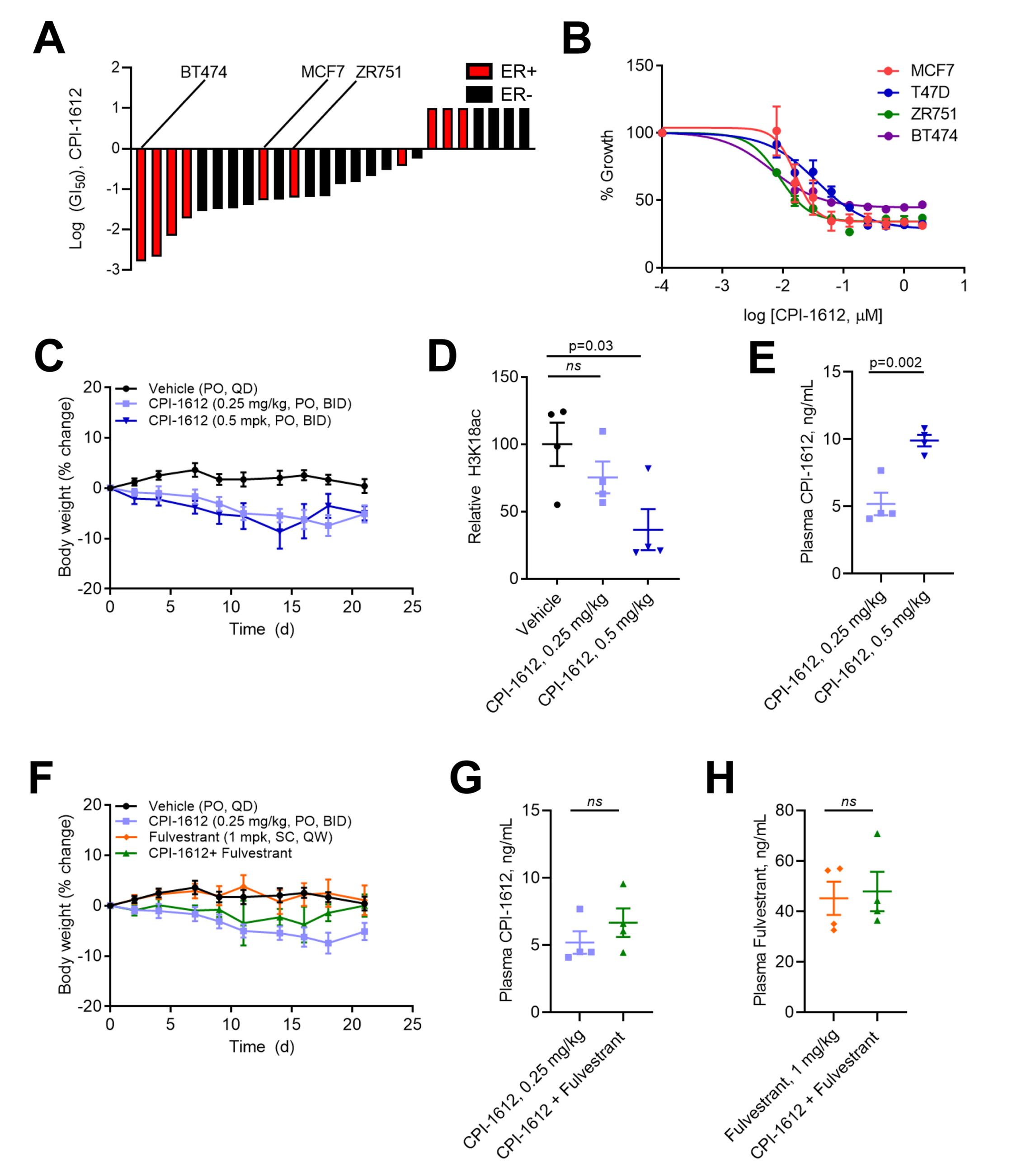

Supplement: S1 Fig — A, Pooled, barcoded cell lines were treated with a dose titration of CPI-1612 for 4 days, and growth inhibition was calculated using depletion of cell line barcodes relative to initial representation. The concentration at which growth was inhibited to 50% of the untreated cells (GI50) was calculated. Red, ER+ cell lines; black: ER- cell lines. B, As in Fig 1A, except cells were treated in media containing charcoal-stripped serum and added estradiol. Error bars represent SD of 2 replicates. C, Change in body weight relative to dosing initiation during treatment with CPI-1612. Error bars represent the SEM at each time point. Data are expressed as H3K18ac signal normalized to total H3 signal in tumor samples from each animal, with mean and SEM shown. P-values were calculated using an unpaired student’s t-test. ns: p>0.05. D, Relative H3K18ac in xenografted MCF7 cells at the endpoint of the study described in Fig 1B. E, Plasma concentration of CPI-1612 at study endpoint. Data are expressed as mean and SEM across 4 mice, and p-value was calculated using an unpaired student’s t-test. F, Change in body weight relative to dosing initiation during treatment with CPI-1612 or Fulvestrant. Error bars represent the SEM at each time point. G, Plasma concentration of CPI-1612 at study termination for single agent or combination treatment. Data are expressed as mean and SEM across 4 mice, and p-value was calculated using an unpaired Student’s t-test. ns: p>0.5. H, Plasma concentration of Fulvestrant at study termination for single agent or combination treatment. Data are expressed as mean and SEM across 4 mice, and p-value was calculated using an unpaired student’s t-test. ns: p>0.05. (TIF) [file pone.0262378.s001.tif]

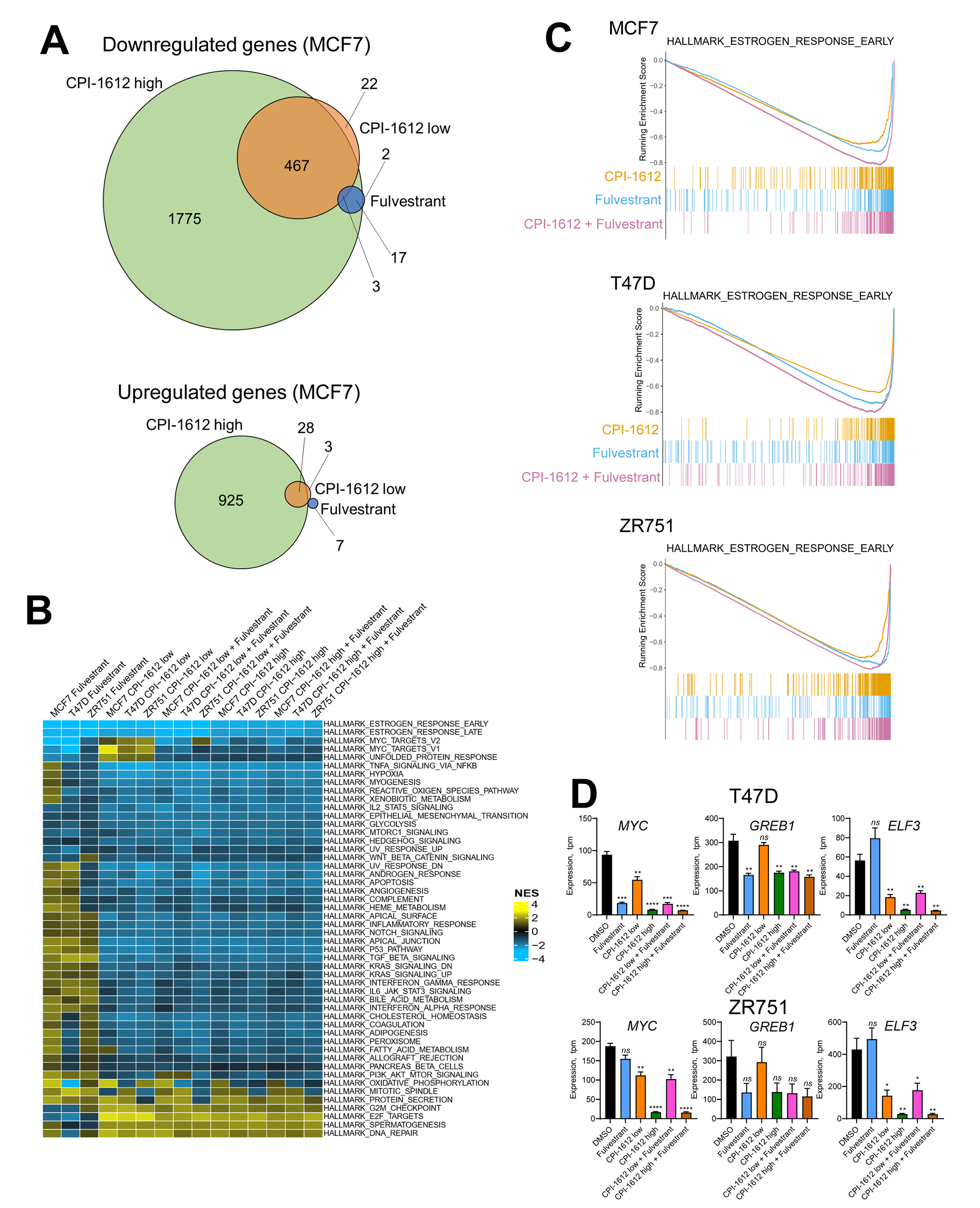

Supplement: S2 Fig — A, Venn diagram of genes down- or upregulated by CPI-1612 or Fulvestrant treatment in MCF7 as in Fig 2. Numbers indicate genes down- or upregulated at least 1.5-fold with an adjusted p-value <0.05 in DESeq2 comparisons to DMSO-treated cells. B, Summary of GSEA against Hallmark genesets for MCF7, T47D, and ZR751 cells treated with the indicated compounds as described in Fig 2. C, Enrichment plots for GSEA of RNA-seq data for the HALLMARK_ESTROGEN_RESPONSE_EARLY geneset in MCF7, T47D, or ZR751 cells treated with CPI-1612 low, Fulvestrant, or CPI-1612 low + Fulvestrant as described in Fig 2. D, Example of differential gene regulation by CPI-1612 and Fulvestrant in T47D and ZR751 cells as described in Fig 2D. Values represent the mean and SEM for 3 replicates. P-values were calculated by unpaired student’s t-test (*: p<0.05; **:p<0.01;***:p<0.001;****:p<0.0001; ns: not significant). P-values can be found in S6 Data. (TIF) [file pone.0262378.s002.tif]

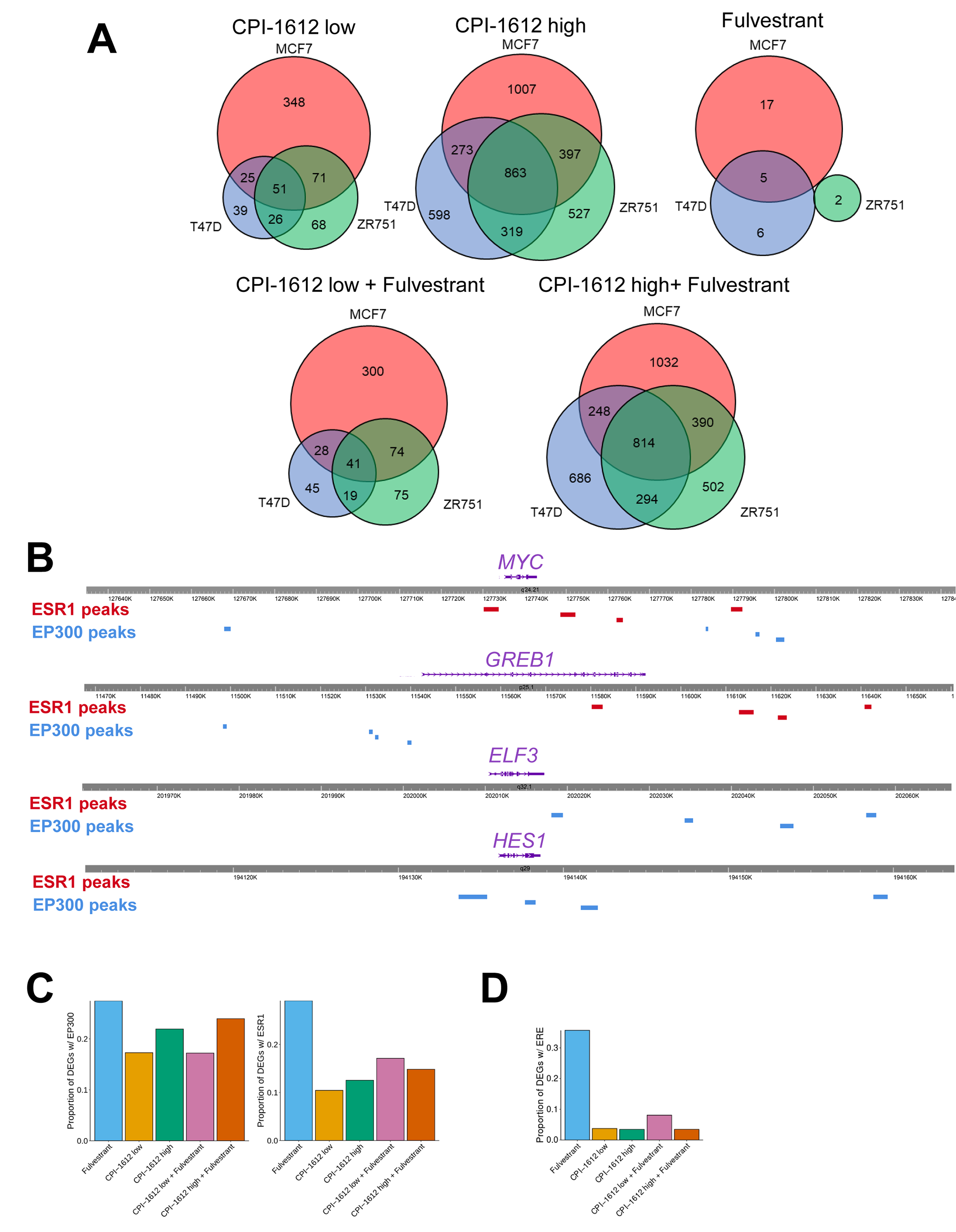

Supplement: S3 Fig — A, Venn diagrams of genes downregulated at least 1.5-fold in MCF7, T47D, and ZR751 upon treatment with the indicated conditions as in Fig 2. B, Genomic tracks for the indicated genes showing annotated peaks for ESR1 or EP300 as colored bars. Note that ELF3 and HES1 do not have annotated ESR1 peaks. C, Fraction of differentially expressed genes from the indicated treatments that were annotated by ChIPseeker as the nearest gene to an EP300 (left panel) or ESR1 (right panel) peak. D, Fraction of differentially expressed genes with an annotated Estrogen Response Element (ERE). (TIF) [file pone.0262378.s003.tif]

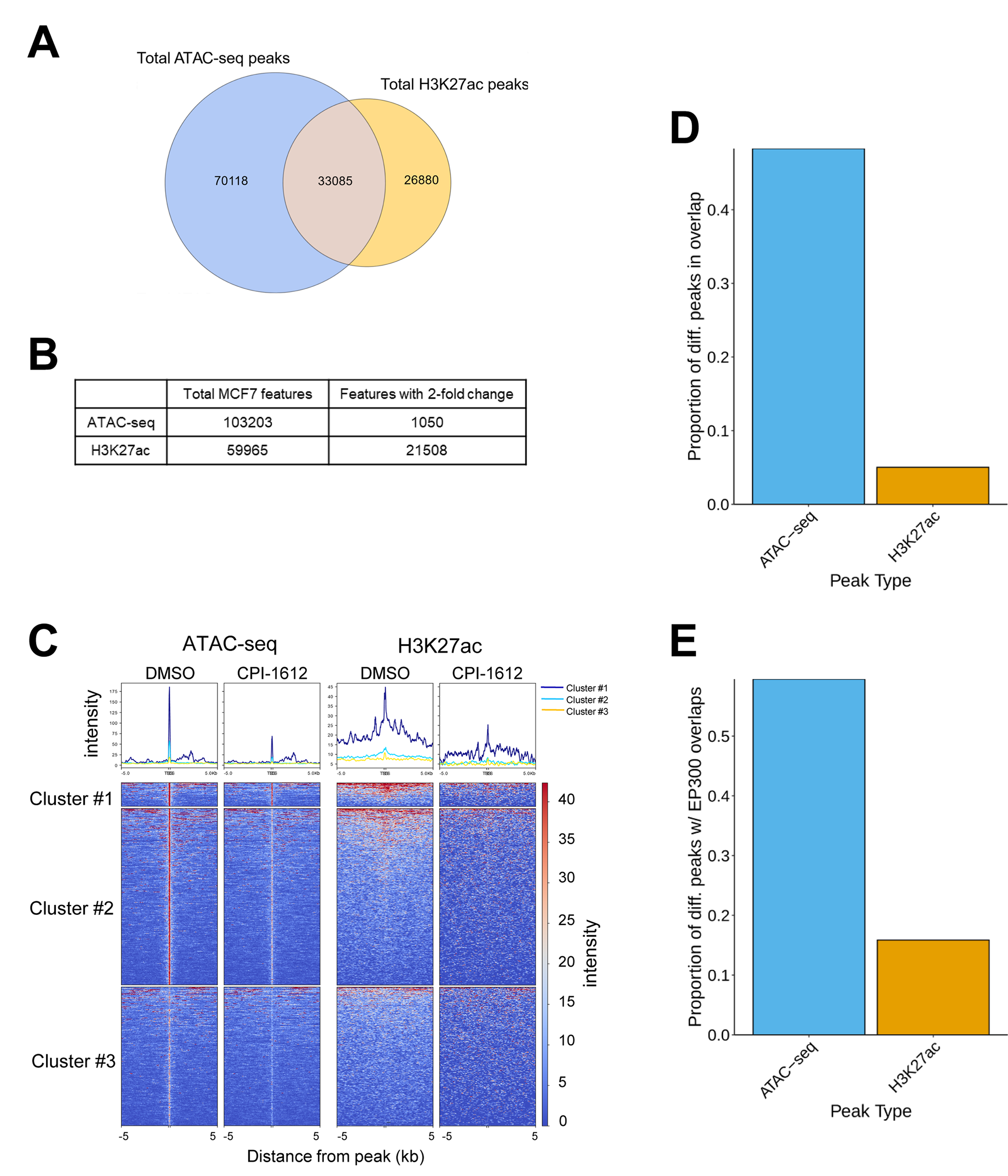

Supplement: S4 Fig — Comparison of changes in chromatin accessibility and H3K27ac after treatment with CPI-1612. A, Venn diagram showing the overlap of all ATAC-seq peaks H3K27ac peaks. B, Table summarizing total ATAC-seq and H3K27ac peaks, and peaks with a 2-fold change upon CPI-1612 treatment. C, Heatmap of differential ATAC-seq peaks showing that peaks that show the largest change in ATAC-seq signal (k-means cluster #1) are most likely to show a reduction in H3K27ac signal. D, Fraction of differential ATAC-seq peaks that are also differential H3K27ac peaks (blue bar), and fraction of differential H3K27ac peaks that are also differential ATAC-seq peaks (orange bar). E, Fraction of differential ATAC-seq peaks and differential H3K27ac peaks that are also occupied by EP300. (TIF) [file pone.0262378.s004.tif]

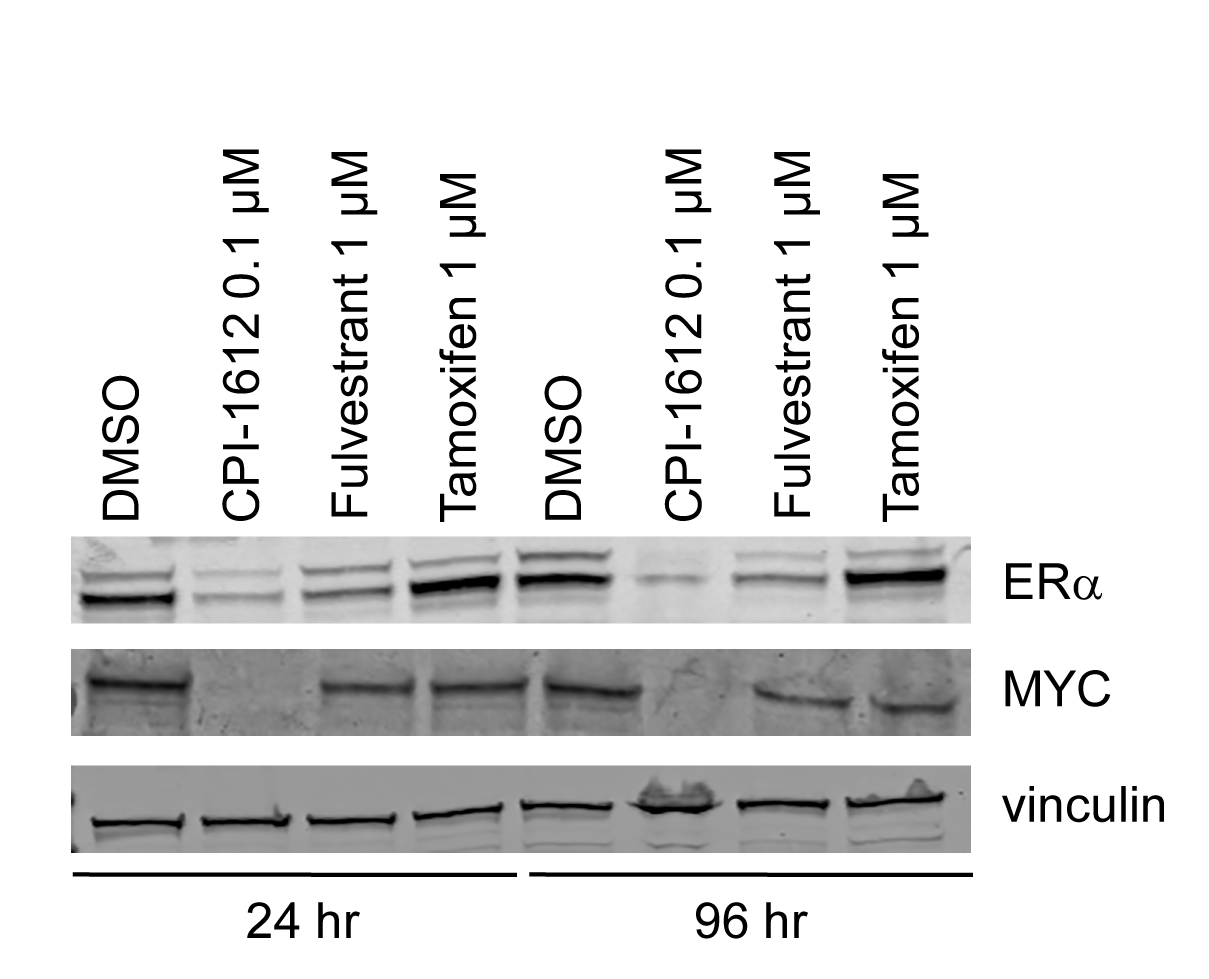

Supplement: S5 Fig — Western blot of MCF7 cells treated with DMSO, CPI-1612 (0.1 μM), Fulvestrant (1 μM), or Tamoxifen (1 μM) for 24 or 96 hours and probed with the indicated antibodies. (TIF) [file pone.0262378.s005.tif]

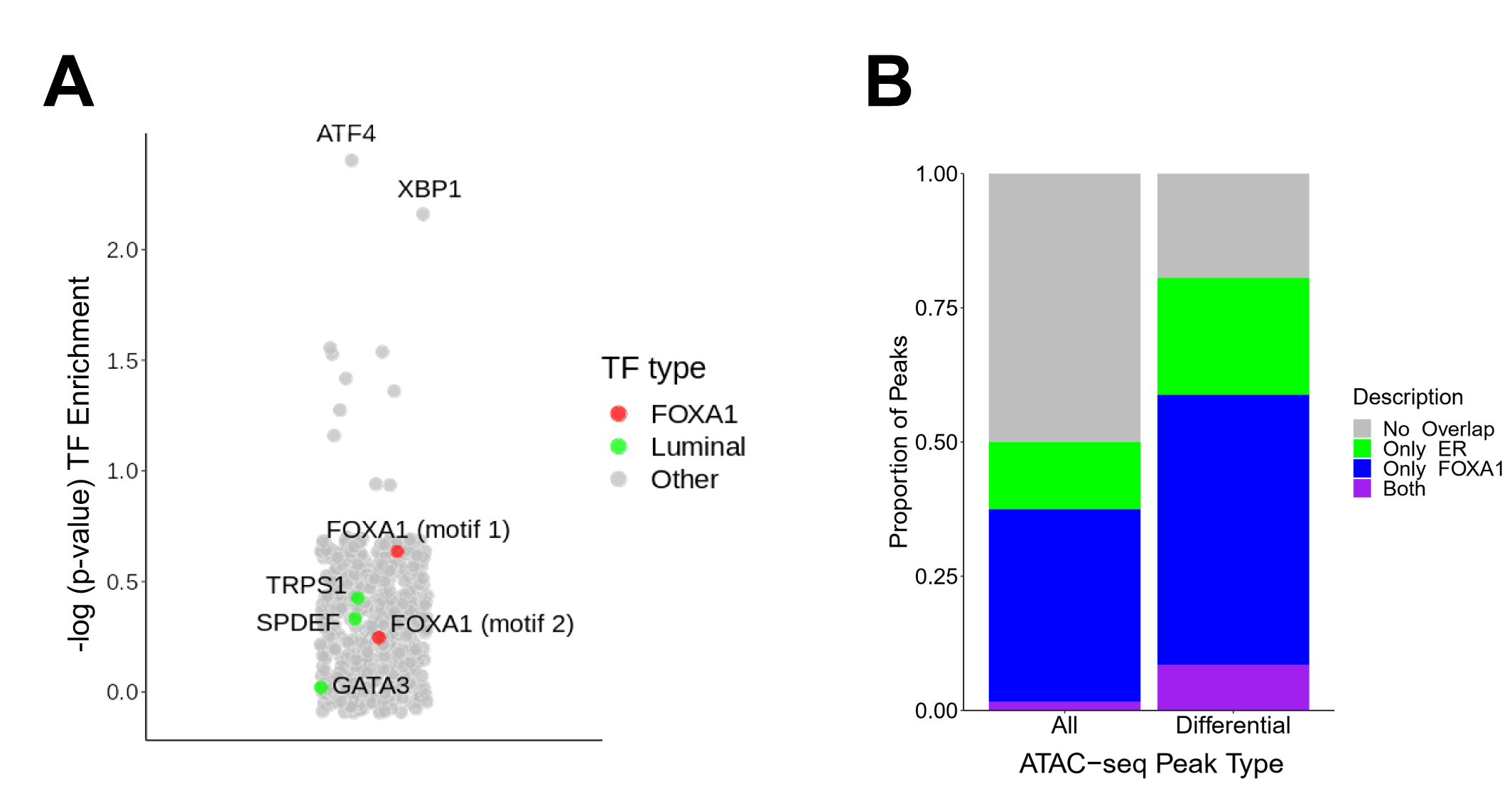

Supplement: S6 Fig — A, HOMER motif search of differential H3K27ac peaks upon CPI-1612 treatment. Binding sites for FOXA1 and luminal specific TFs are not enriched in differential H3K27ac peaks relative to all H3K27ac peaks. B, Overlap of transcription factor (TF) binding with all identified ATAC-seq peaks or those peaks that showed at least a 2-fold reduction in signal after CPI-1612 treatment. ER and FOXA1 data are as described in Fig 4C. (TIF) [file pone.0262378.s006.tif]

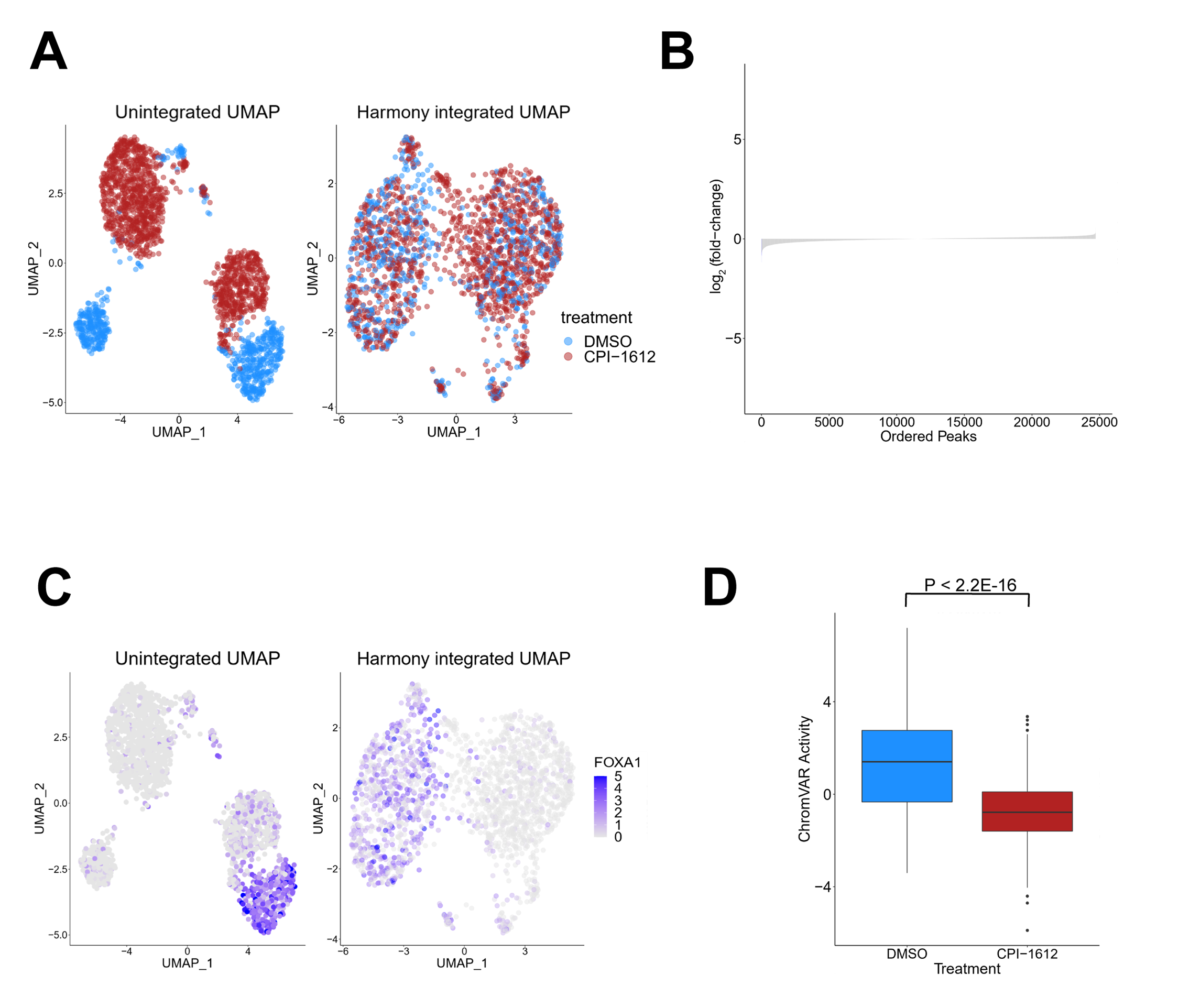

Supplement: S7 Fig — A, UMAP dimensionality reduction plot of scATAC-seq data colored by treatment for both unintegrated cells and data integrated using Harmony. B, Waterfall plot of peaks identified from scATAC-seq data ranked by log2 (fold-change) for CPI-1612 relative to DMSO. Blue, peaks reduced by at least 2-fold. C, UMAP plot as in A, colored by predicted FOXA1 activity based on ChromVAR analysis. D, Quantification of predicted FOXA1 activity for DMSO and CPI-1612 treated cells. Boxplots depict median and range of FOXA1 (MA0148.4 motif) activity; p-value was calculated with the Mann-Whitney U test. (TIF) [file pone.0262378.s007.tif]

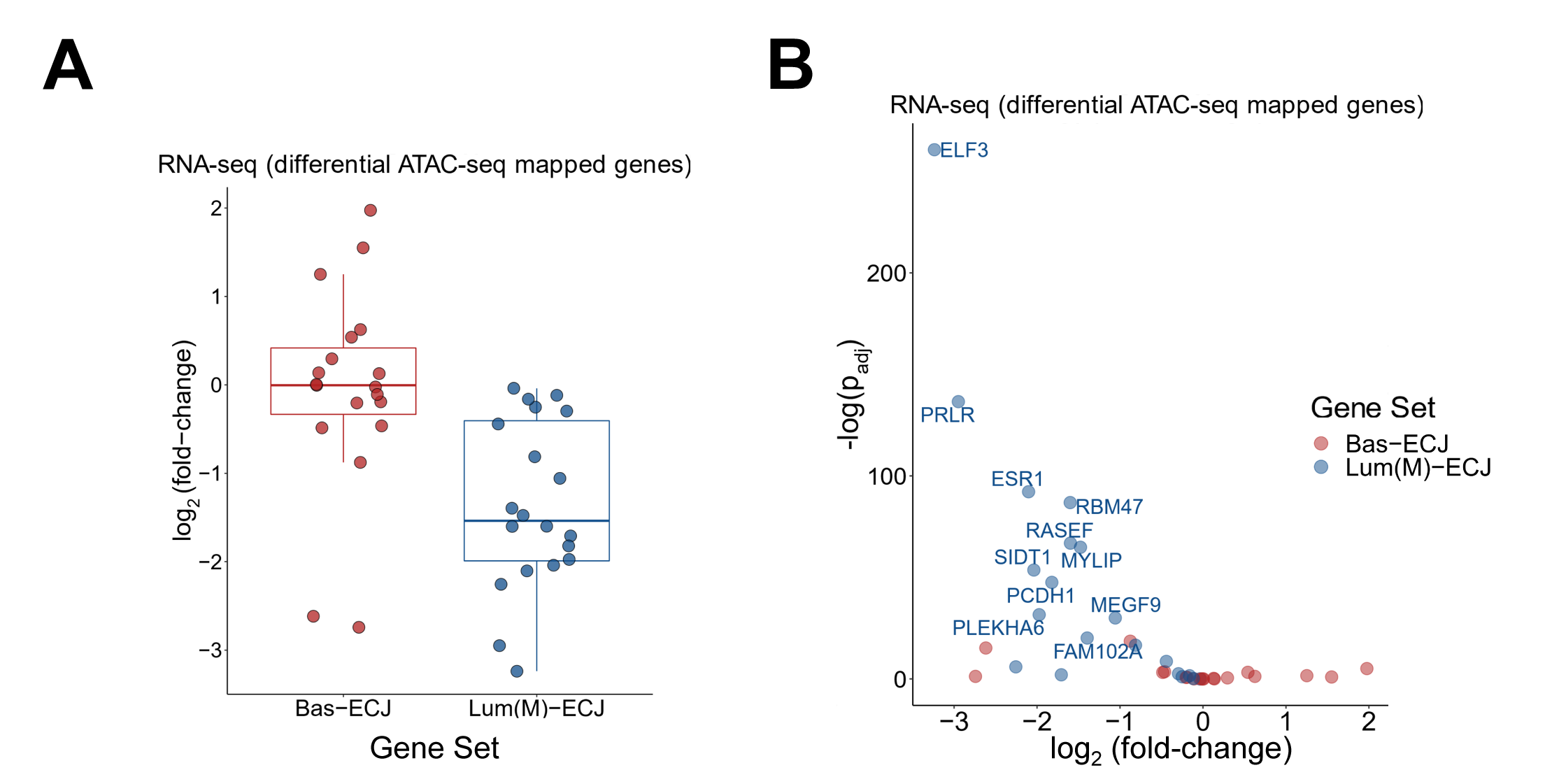

Supplement: S8 Fig — A, Differential expression of genes in the Bas-ECJ or Lum(M)-ECJ gene sets upon treatment with CPI-1612. B, Volcano plot of gene expression changes as described in B. (TIF) [file pone.0262378.s008.tif]

kDa

75 →

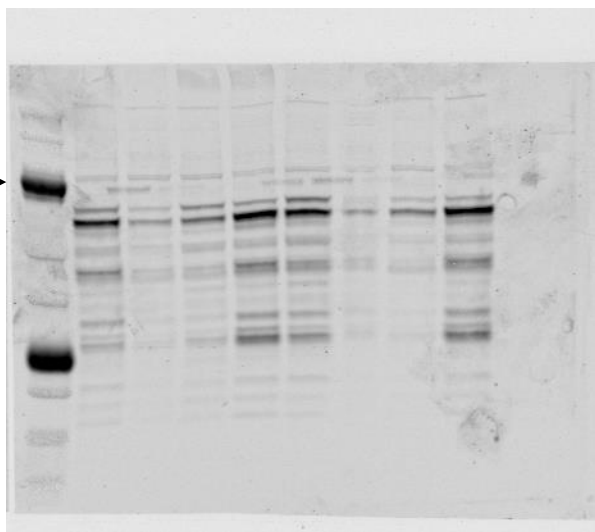

ER $\alpha$   
Expected 66 kDa

kDa

50 →

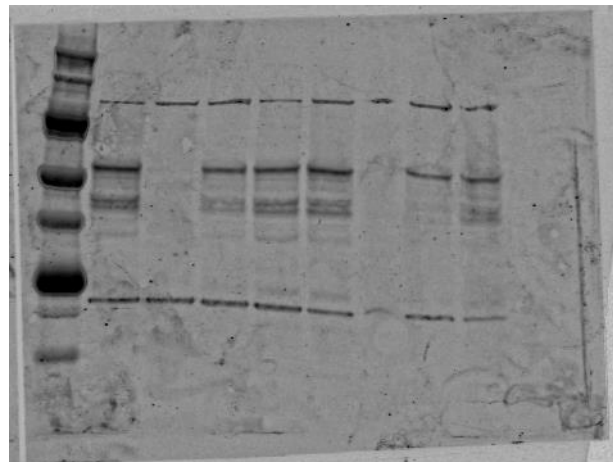

MYC  
Expected 48 kDa

kDa

100 →

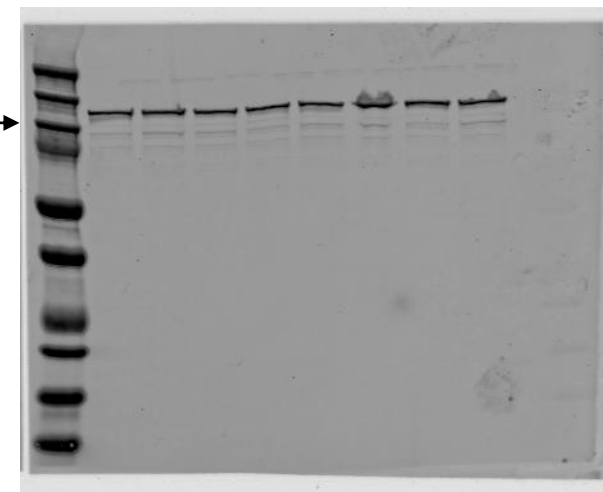

Vinculin  
Expected 117 kDa

Supplement: S1 File — (PDF) [file pone.0262378.s020.pdf]
